# PCR-Free Enrichment of Mitochondrial DNA from Human Blood and Cell Lines for High Quality Next-Generation DNA Sequencing

**DOI:** 10.1371/journal.pone.0139253

**Published:** 2015-10-21

**Authors:** Meetha P. Gould, Colleen M. Bosworth, Sarah McMahon, Sneha Grandhi, Brian T. Grimerg, Thomas LaFramboise

**Affiliations:** 1 Department of Genetics and Genome Sciences, Case Western Reserve University, Cleveland, Ohio, United States of America; 2 Center for Global Health and Disease, Case Western Reserve University, Cleveland, Ohio, United States of America; 3 Case Comprehensive Cancer Center, Case Western Reserve University, Cleveland, Ohio, United States of America; 4 Department of Electrical Engineering and Computer Science, Case Western Reserve University, Cleveland, Ohio, United States of America; 5 Genomic Medicine Institute, Lerner Research Institute, Cleveland Clinic Foundation, Cleveland, Ohio, United States of America; Ben-Gurion University of the Negev, ISRAEL

## Abstract

Recent advances in sequencing technology allow for accurate detection of mitochondrial sequence variants, even those in low abundance at heteroplasmic sites. Considerable sequencing cost savings can be achieved by enriching samples for mitochondrial (relative to nuclear) DNA. Reduction in nuclear DNA (nDNA) content can also help to avoid false positive variants resulting from nuclear mitochondrial sequences (numts). We isolate intact mitochondrial organelles from both human cell lines and blood components using two separate methods: a magnetic bead binding protocol and differential centrifugation. DNA is extracted and further enriched for mitochondrial DNA (mtDNA) by an enzyme digest. Only 1 ng of the purified DNA is necessary for library preparation and next generation sequence (NGS) analysis. Enrichment methods are assessed and compared using mtDNA (versus nDNA) content as a metric, measured by using real-time quantitative PCR and NGS read analysis. Among the various strategies examined, the optimal is differential centrifugation isolation followed by exonuclease digest. This strategy yields >35% mtDNA reads in blood and cell lines, which corresponds to hundreds-fold enrichment over baseline. The strategy also avoids false variant calls that, as we show, can be induced by the long-range PCR approaches that are the current standard in enrichment procedures. This optimization procedure allows mtDNA enrichment for efficient and accurate massively parallel sequencing, enabling NGS from samples with small amounts of starting material. This will decrease costs by increasing the number of samples that may be multiplexed, ultimately facilitating efforts to better understand mitochondria-related diseases.

## Introduction

Mitochondria are involved in fundamental cellular processes including generating ATP for cellular energy, storing calcium for cell signaling, and mediating cell growth and death. A number of human diseases are tied to dysregulation of mitochondrial function. Dysregulation is often the result of DNA-level mutations affecting mitochondrial proteins [1], which may be encoded in either the nuclear genome or the mitochondrial genome. The two genomes have different codon usage and separate translational machinery. Detecting DNA-level variants in the mitochondrial genome presents unique challenges. Although the genome is much smaller than its nuclear counterpart–some 16.5 kilobases as compared to 3.2 gigabases–it is present at hundreds to thousands of copies per cell. As such, a mutation may be present in a very low percentage of mtDNA copies, in contrast to nuclear variants which are normally present in 0%, 50%, or 100% of the cell’s nuclear genome (nDNA) copies. Low-level mtDNA variants were difficult or impossible to detect using traditional Sanger sequencing, but the emergence of “next-generation” sequencing (NGS) now makes very sensitive detection possible [2]. However, variant calling can be confounded by the presence of nuclear mitochondrial DNA sequences (numts). Numts are tracts of nDNA that are near or perfect matches to the mtDNA sequence. These numts can give rise to false positive variant calls when mistaken for mtDNA, as their small deviations from the mitochondrial reference sequence are misidentified as mitochondrial variants. Furthermore, if the mitochondrial genome is of primary interest in a NGS experiment, any nuclear reads add unnecessary cost and effort. The added cost can be considerable since total genomic DNA is approximately 99.8% nuclear. For economic efficiency, and to avoid numt-induced false positives, it is important to enrich the DNA sample for mtDNA, either through mitochondrial isolation or mtDNA amplification.

Differential centrifugation (DC) is commonly used to isolate mitochondria. Typically, the method entails cell lysis followed by serial gradient centrifugations, the first to remove the heavier cellular components and the second to extract the mitochondrial organelles. DC is relatively straightforward and allows for abundant mitochondrial isolates. However, due to the harsh nature of high-speed centrifugation, disruptions of the nuclear and the mitochondrial membranes can lead to nDNA contamination and less robust mtDNA enrichment. As an alternative, recent studies [3, 4] describe isolation of mitochondria from cell lysate using magnetic beads coupled to TOM22 antibodies. These studies report that the magnetic bead (MB) isolation approach yields whole mitochondria with intact membrane machinery, and less contamination as compared to mitochondrial isolation using DC.

Amplification of the mitochondrial genome is also frequently used to enrich for mtDNA sequences. Amplification can be performed either using multiple primers, resulting in smaller mtDNA fragments, or using one to two primer pairs to obtain the genome in large fragments via long-range PCR. For the purposes of NGS, amplification is most commonly performed using long-range PCR [5, 6]. While long-range PCR results in high levels of mtDNA enrichment, each cycle of amplification can induce false mutations owing to polymerase errors. These errors are difficult to distinguish from true mutations in the downstream NGS reads. Polymorphisms or mutations in the primer region also may interfere with the PCR process, distorting allele frequencies in the downstream amplicons. Furthermore, long-range PCR requires a relatively large amount of template DNA, which may be challenging to obtain depending on the experiment. For example, some blood fractions harbor very low concentrations of DNA.

In this paper, we compare various mitochondrial isolation and mtDNA enrichment procedures to perform NGS of human mitochondrial DNA from blood components and cell lines. We also assess the use of an exonuclease digest, which we hypothesized would preferentially deplete nDNA since the digest cleaves nucleotides from the ends of the DNA strands. We use real-time quantitative PCR (qPCR) and NGS results as enrichment metrics. For NGS, we have adopted Illumina’s Nextera XT library preparation, allowing for sample barcoding for multiplex sequencing from only 1 ng of template DNA per sample.

## Materials and Methods

### Ethics statement

Whole blood was collected from healthy volunteers, under IRB-approved informed written consent (University Hospitals Case Medical Center IRB Approval # 12-09-07). The University Hospitals Case Medical Center Institutional Review Board specifically approved this study.

### Isolation of mitochondria from cell lines

COLO 829 and COLO 829BL (tumor/normal matched) cell lines were purchased from ATCC. DC isolation was performed using the Qproteome kit (Qiagen), and MB isolation with TOM22 antibody bound to MACS magnetic beads was performed using the Mitochondrial Isolation Kit (Miltenyi Biotech), both according to manufacturers’ protocols. MtDNA was extracted from pellet using DNeasy Blood and Tissue kit (Qiagen), eluted using smaller volumes.

### Whole blood fractionation

Twenty-five milliliters (mls) of whole blood was collected from healthy volunteers, under IRB-approved informed consent (University Hospitals Case Medical Center IRB Approval # 12-09-07; the University Hospitals Case Medical Center Institutional Review Board specifically approved this study), into vacutainers with EDTA to inhibit coagulation. Samples were then separated into plasma, buffy layer, peripheral blood mononuclear cells (PBMCs) and erythrocytes (RBCs) fractions by density gradient centrifugation using Ficoll-Paque Plus (GE Healthcare) under their supplied protocol within an hour of blood collection. The intact mitochondria were then isolated from these fractions.

### Modified DC isolation for blood

We used a modified protocol from the Qproteome kit (Qiagen), omitting the lysis step then using 2 mls of the separated fractions and centrifuging at 1,000 × g for 10 minutes at 4°C. We transferred the supernatant to a clean tube and centrifuged again at 6,000 × g for 10 minutes at 4°C. The pellet was resuspended in 1 ml mitochondrial storage buffer and centrifuged at 6,000 × g for 20 minutes at 4°C. MtDNA was extracted from pellet as stated above.

### Modified MB isolation for blood

We used a modified protocol from the Mitochondrial Isolation kit (Miltenyi Biotec) to isolate intact mitochondria from blood fractions. The subcellular organelle was magnetically labeled with the anti-TOM22 microbeads immediately after centrifugation by incubating 2.5 mls of each of the separated fractions with 75 μl of microbeads in a total volume of 15 mls with separation buffer for 60 minutes at 4°C on a nutator. The pull down was completed as per the Miltenyi Biotec protocol. MtDNA was extracted from pellet as stated above.

### Real-Time quantitative PCR

Following double-stranded DNA quantification using Quant-iT PicoGreen dsDNA Assay kit (Life Technologies), relative and absolute copy numbers were determined by qPCR using a 7300 Real Time PCR system (Life Technologies). We used human RNAse P Taqman Copy Number Reference Assay, Taqman 18S Endogenous Control, and Taqman MT-ND1 Gene Expression Assay (Life Technologies). The Minimum Information for Publication of Quantitative Real-Time PCR Experiments (MIQE) guidelines [7] were adhered to. Each 96-well plate included a *ρ* ^0^ negative control consisting of human DNA devoid of mtDNA [8] (RhoZero). Standard curves for both mtDNA and nDNA assess primer PCR efficiency with slopes between -3.6 and -3.1. Quantification for copy number was based on standard curves determined from a serial dilution of pooled female human genomic DNA (Promega). The relative standard curve method was used to calculate target mtDNA copy abundance in each sample normalized to the nDNA target. Each reaction produced the average threshold cycle (C_t_) amounts for mtDNA and nDNA. To calculate ΔC_t_ we used the average C_t_ value for mtDNA less the average C_t_ value for nDNA. The amount of mtDNA relative to nDNA is determined by 2^-ΔCt^.

### Long Range PCR of the mitochondrial genome

MtDNA was amplified using long range PCR resulting in two long overlapping fragments of ~8.5 kb each. Specifically, primer sequences used were AAATCTTACCCCGCCTGTTT (forward) and AATTAGGCTGTGGGTGGTTG (reverse) for one amplicon, and GCCATACTAGTCTTTGCCGC (forward) and GGCAGGTCAATTTCACTGGT (reverse) for the other. Long range PCR was completed using Advantage GC Genomic LA Polymerase Mix (Clontech), which includes a proofreading enzyme and a hot-start antibody. Long range PCR reaction conditions were 94°C for 1 min, 30 cycles of 94° for 30 seconds, 56°C for 30 seconds, 72°C for 9 minutes with a final extension of 72°C for 5 minutes. PCR products were run on a 0.8% agarose gel, then the expected 8.5 Kb fragments were excised. DNA was extracted using a QIAEX II Gel Extraction Kit (Qiagen). Quantification was assessed by Nanodrop.

### Exonuclease digest

Digestion of linear DNA was performed using Plasmid Safe ATP-dependent DNAse (EpiBio). Manufacturer’s protocol was followed, except that all volumes were halved save that of ATP. Total input DNA varied between 2 to 50 ng, which was incubated for 30 minutes.

### Isolation of cell-free DNA

PureLink Viral RNA/DNA kit (Life Technologies) was used to isolate cell-free DNA (cfDNA) as per manufacturer’s instructions.

### Library preparation and NGS

For each sample, 1 ng of DNA was indexed using the Nextera XT library preparation kit (Illumina). During the Nextera procedure, after the tagmentation, amplification, and PCR cleanup steps, fragment size distribution was assessed using the Bioanalyzer (Agilent) and total DNA concentration was determined using the PicoGreen assay. Inputs were normalized accordingly. The remainder of the library preparation was performed as per manufacturer protocols. In each run, a *ρ* ^0^ sample was included as a negative control. DNA sequencing was performed on the Illumina MiSeq, generating paired-end 150 bp reads.

### NGS data processing

From the.fastq files, reads were sorted into individual files based on their adapter sequences. Adapter sequences were automatically removed and quality trimming performed by the Illumina software, followed by alignment to a modified human genome build hg19 using BWA [9] version 0.7.5a-r405 with the "sampe" option. Hg19 was modified by removing the original chrM and replacing it with the revised Cambridge Reference Sequence (rCRS; NC_012920.1) [10]. Samtools version 0.1.18 was used to sort and index the bam files. The Picard (version 1.93) tools AddOrReplaceReadGroups (with options SORT_ORDER = coordinate and VALIDATION_STRINGENCY = SILENT) and MarkDuplicates (with options CREATE_INDEX = true, VALIDATION_STRINGENCY = SILENT, ASSUME_SORTED = true, and REMOVE_Duplicates = true) were applied to sort the .sam file and remove any PCR duplicates. At this stage the number of reads mapping to the mitochondrial genome and the nuclear genome were tallied to compute enrichment in mtDNA. Version 2.6–5 of the Genome Analysis Toolkit (GATK) [11] was used to ensure quality variant calls. Following best practices, "walkers" used (in order) were: RealignerTargetCreator with the-L chrM:1–16569 option specified; IndelRealigner; Picard's FixMateInformation; BaseRecalibrator with dbSNP version 137; PrintReads with the-BQSR option; UnifiedGenotyper with options-glm BOTH (genotype-likelihoods model calling both SNPs and indels)-stand_call_conf 50.0-stand_emit_conf 10.0-dcov 1000-L chrM:1–16569, and the-D option; and VariantFiltration using filters HARD_TO_VALIDATE (MQ0 > = 4 && ((MQ0 / (1.0 * DP)) > 0.1), LowCoverage (DP<5), LowQuality (QUAL<100.0), LowQD (QD<1.5), and StrandBias (FS > 40.0). The resulting.vcf file was then used to identify variants from those that passed the filters.

Overall, PCR duplicate rate ranges from <0.1% to 15.8% for all mapped reads (mean 3.1%), and from 0% to 15.7% for mtDNA mapped reads (mean 5.5%). PCR duplicate information is summarized in S1 Table.

### Expected proportion of mtDNA reads in NGS data from unenriched total genomic DNA

The theoretical unenriched proportion of reads expected to come from mtDNA is computed assuming approximately 660 copies of the mitochondrial genome per cell:

$$\%\text{mitochondrial reads}\;\approx \frac{660\;\text{copies}\;\times 16569\;\text{bases}}{\left(660\;\text{copies}\;\times 16569\;\text{bases}\right)+\left(2\;\text{copies}\;\times 3.2\;\text{billion bases}\right)}\approx 0.2\%.$$

Here the estimated 660 copies is derived from qPCR of pooled female human genomic DNA from blood (Promega), where the mitochondrial *MT-ND1* locus was estimated to have approximately 330 times more copies than the (two-copy) RNAse P locus (S2 Table). The same qPCR experiment was performed using one of our normal blood samples, with very similar results (S2 Table).

### Sanger sequencing validation

NGS library-prepared products were sequenced using BigDye Terminator (Life Technologies) chemistry on an ABI3730 automated DNA sequencer (Life Technologies).

### Data availability

Data is freely available for download from Dryad at http://dx.doi.org/10.5061/dryad.bq4t8

## Results

### Measuring enrichment in cell lines using qPCR

We first tested the isolation procedures on the metastatic melanoma cell line COLO 829 and its matched normal lymphoblast line COLO 829BL [12]. Before proceeding to next-generation sequencing, we assessed enrichment using qPCR. Since *MT-ND1* is present at hundreds to thousands of copies per cell, using a two-copy gene as a comparator limits precision in measuring relative abundances of mtDNA across samples. 18S is present at some 300–400 copies per cell [13], closer to mitochondrial genome abundance. Using 18S as the nDNA reference allows more precise assessment of relative mitochondrial enrichment between samples, while using the RNAse P gene allows some estimate of per-cell mtDNA copy number since *RPPH1* is present at two copies per cell.

**Fig 1:**
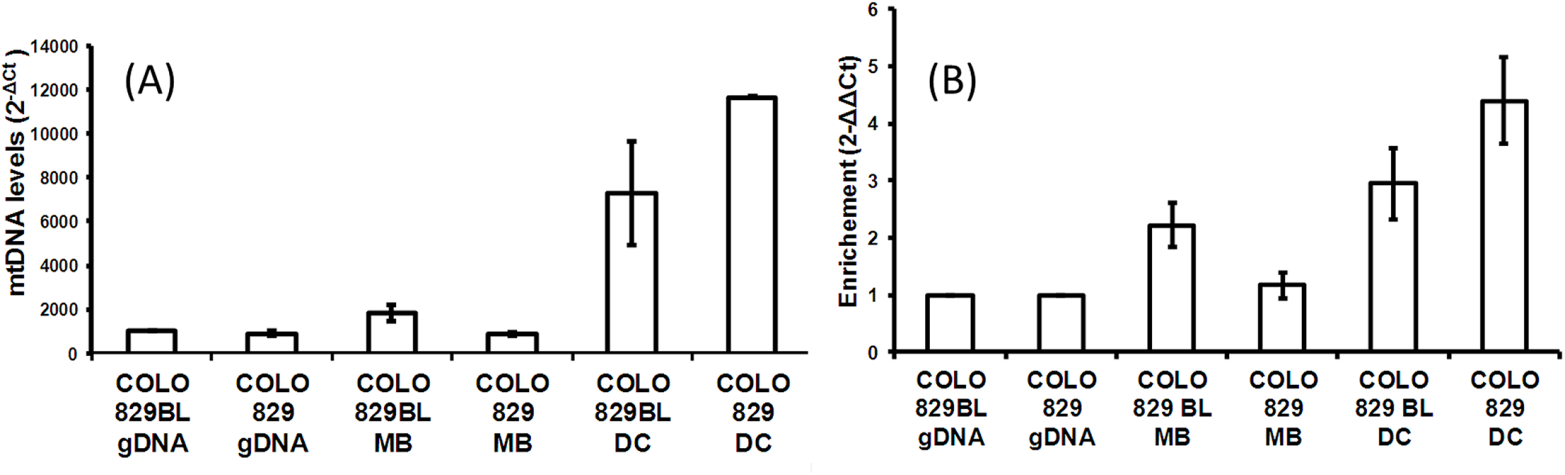
qPCR assessment of mtDNA enrichment for DC and MB isolation in cell lines. Here the mitochondrial genome is represented using the *MT-ND1* gene locus. As nuclear comparators, the genes *RPPH1* **(A)** or 18S **(B)** are used. Error bars represent ± one standard deviation from the mean. In **(A)**, the 2^-ΔC^_t_ vertical axis represents the estimated amount of *MT-ND1* as compared to RNAse P. In **(B)**, the vertical axis is scaled relative to the baseline total genomic DNA (gDNA) samples (COLO 829BL gDNA and COLO 829 gDNA). Note that, in both cases, DC isolation yields the most robust enrichment.

Real-time qPCR comparing mtDNA abundance using the RNAse P gene (Fig 1A) showed considerable enrichment for cells subjected to DC as compared to total genomic DNA baseline, for both COLO 829 and COLO 829BL. The MB COLO 829BL isolate also showed enrichment, albeit to a lesser degree. Using 18S as the nDNA representative (Fig 1B) shows 1.2- to 2.2-fold enrichment for MB isolation and 3- to 4.4-fold enrichment for DC isolation. Since our goal is to assess relative mtDNA enrichment between samples, for the remainder of this study we use 18S as the nDNA representative since, as discussed above, measurements of its relative abundance are likely to be more accurate.

### Next-generation sequencing of cell lines

**Fig 2:**
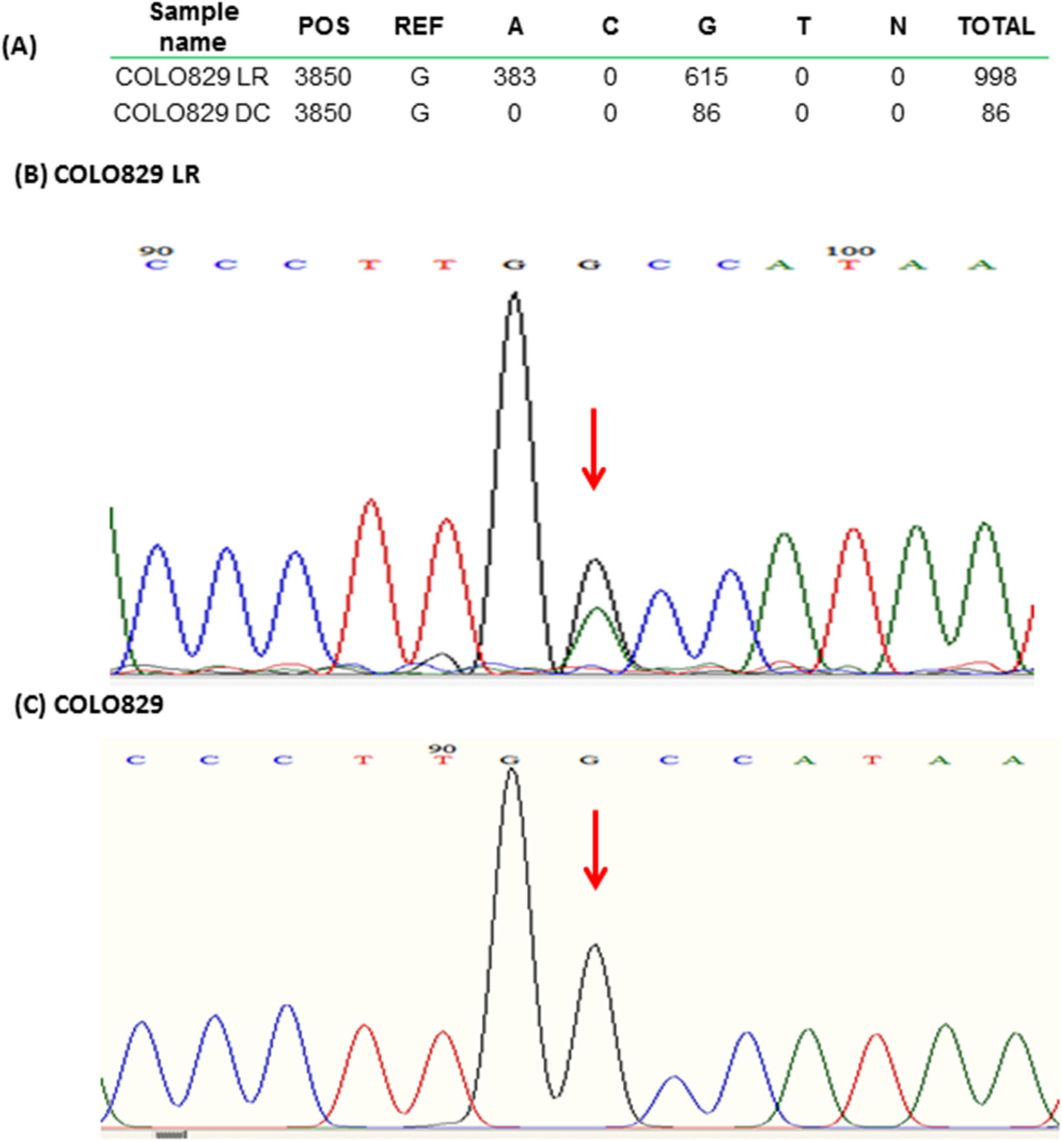
False heteroplasmy call and Sanger sequencing validation. (A) Table of showing counts, by allele, of all reads covering position 3850 counts. The non-PCR amplified sample (COLO 829 DC) is purely homoplasmic reference (G), but the LR sample shows G/A heteroplasmy. Sequencing traces for the COLO 829 LR (B) and COLO 829 gDNA(C) confirm that the PCR step did induce the false heteroplasmy. Position 3850 is indicated with the rectangle and arrow in both panels, showing heteroplasmy (black and green peaks colocalizing) in the LR sample.

We next performed NGS on the cell isolates (NGS Run 1). MtDNA enrichment can be measured in NGS data as the proportion of reads mapping to mtDNA *vs*. nDNA. Compared to the mtDNA proportion of ~0.2% expected from unenriched total genomic DNA, both MB and DC isolation showed some enrichment for both COLO 829 and COLO 829BL (Table 1), but still had majority residual nDNA. Unsurprisingly, the highest level of enrichment was observed from samples undergoing long-range PCR, with greater than 95% of reads mapping to the mitochondrial genome. However, comparing sequence content from the amplified DNA samples with those not subjected to long-range PCR (LR) revealed artifacts. We found that COLO 829 LR showed significant heteroplasmy at position 3850 that was absent in the unamplified cell line DNA (Fig 2A). Sanger sequencing of the LR DNA sample confirmed the heteroplasmy call and its absence in the original sample (Fig 2B and 2C). Given this defect, we restricted further investigation to the procedures that did not involve PCR amplification before library preparation.

**Table 1:** NGS Summary of Run 1.

Run	Sample	Total Reads	Reads Aligned (% of total)	Reads Aligned to Mitochondrial Genome (% of aligned)
1	COLO 829BL MB	226596	210702 (92.99%)	662 (0.31%)
1	COLO 829BL DC	260994	243342 (93.24%)	1018 (0.42%)
1	COLO 829BL LR	183386	157724 (86.01%)	156215 (99.04%)
1	COLO 829BL MB LR	246128	203366 (82.63%)	201402 (99.03%)
1	COLO 829BL DC LR	202558	171531 (84.68%)	171124 (99.76%)
1	COLO 829 MB	219310	205121 (93.53%)	1024 (0.50%)
1	COLO 829 DC	191944	177542 (92.45%)	10116 (5.70%)
1	COLO 829 LR	209776	175910 (83.86%)	175173 (99.58%)
1	COLO 829 MB LR	192530	165247 (85.83%)	164517 (99.56%)
1	COLO 829 DC LR	172378	149276 (86.60%)	148621 (99.56%)

MB: Magnetic Bead Separation, DC: Differential Centrifugation, LR: long-range PCR

### Exonuclease digest boosts mtDNA enrichment

**Fig 3:**
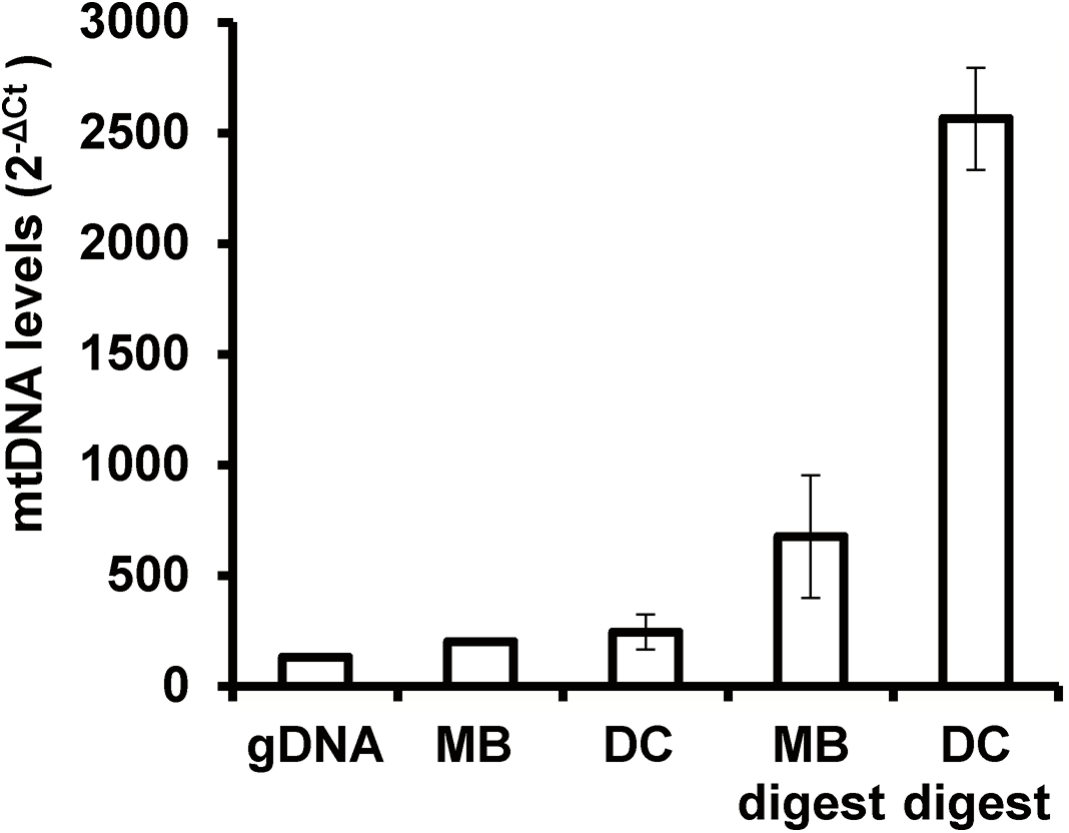
qPCR assessment of mtDNA enrichment from exonuclease digest in COLO829 cell line isolates. Digestion substantially boosts enrichment levels over isolation alone.

To further enrich our samples for mtDNA content, we explored the use of an exonuclease digest. Since the mitochondrial genome is circular, exonuclease should preferentially eliminate (linear) nDNA. qPCR analysis of COLO 829 showed that the digest provided substantial gains in enrichment from both DC and MB isolation in both cell lines (Fig 3). Overall, the best result was obtained using DC isolation followed by exonuclease digest, with enrichment nearly 20-fold over gDNA.

### MtDNA enrichment in blood

**Fig 4:**
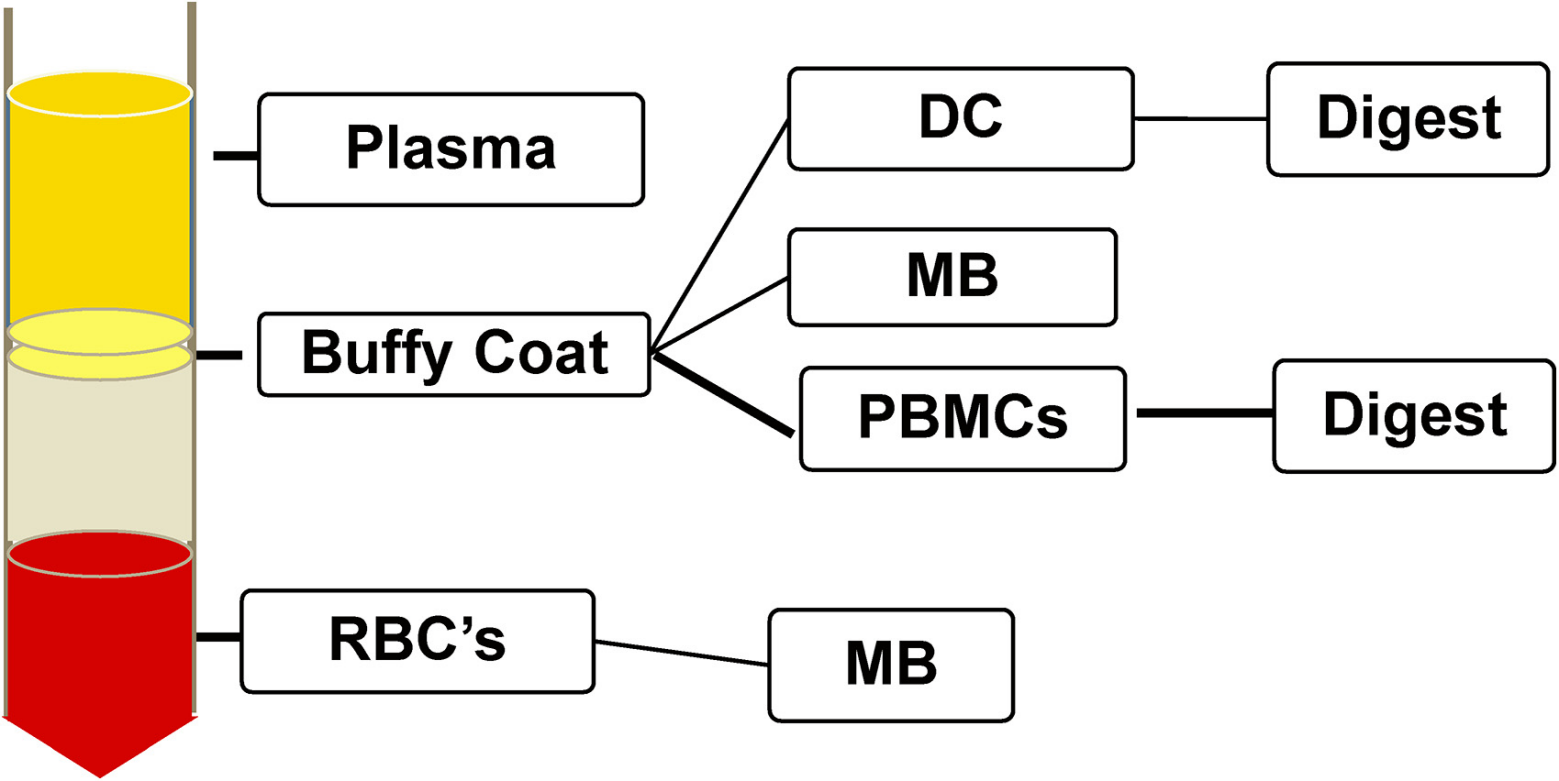
Alternatives for isolating mtDNA from blood. After density gradient centrifugation, each fraction may be subjected to DC or MB isolation. From the resultant DNA, exonuclease digest may also be performed. Note that there was not enough DNA available from plasma isolates or from RBC’s to perform exonuclease digest or NGS.

There are a number of possible procedures for mtDNA enrichment in blood (summarized Fig 4). Isolation may be performed from any of the blood fraction components, and DC or MB isolation (or neither) may be followed by exonuclease digest. We sought to assess all of these enrichment procedures using qPCR and NGS data. As an unenriched baseline, we used total genomic DNA from peripheral blood mononuclear cells (PBMCs).

**Fig 5:**
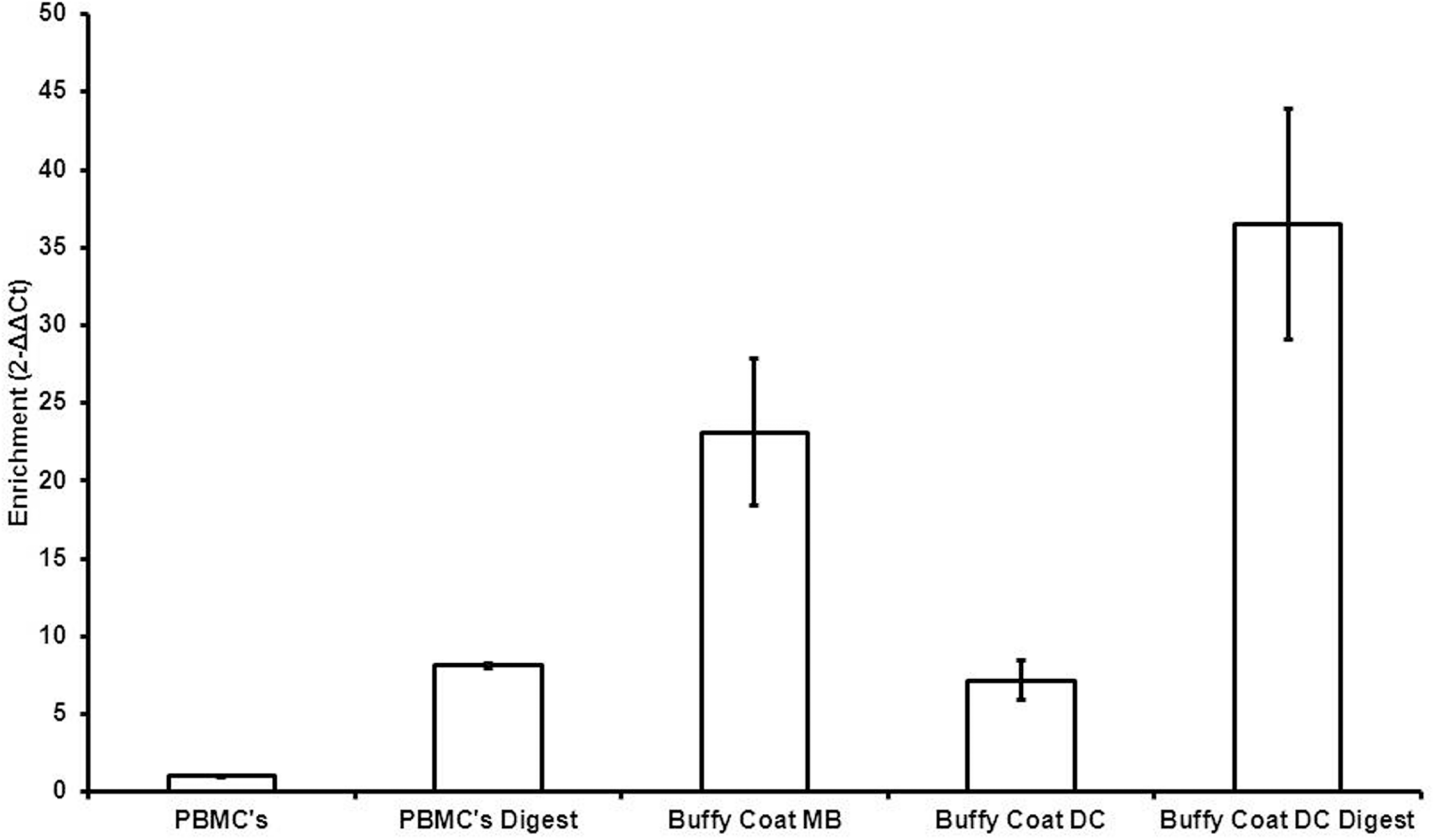
qPCR assessment of mtDNA enrichment from blood sample 1312. The vertical axis is scaled relative to the baseline PBMCs ((*MT-ND1*-*18s*)/PBMCs).

We were unable to obtain adequate quantities of DNA in the plasma isolates and red blood cell (RBC) fractions, as would be expected, but the buffy coat fraction shows strong mtDNA enrichment after DC and MB isolation, according to qPCR (Fig 5). The MB isolation yielded insufficient DNA for exonuclease digest, but enrichment in the DC isolate was substantial following the digest, recapitulating observations from the cell lines.

### Optimizing next-generation sequencing of mtDNA in blood and cells

As a final assessment, we performed two additional NGS runs (2 and 3), each containing a cell line sample (COLO 829 and COLO 829BL, respectively) and blood samples (donors 1309 and 1312, respectively). Given the results from the qPCR experiments, we focused on the buffy coat fraction of the blood samples. The NGS results again showed particularly high levels of enrichment for DC followed by digest (Table 2). The near total absence of mitochondrial reads in the *ρ* ^0^ sample demonstrates that our NGS analysis procedure is not confounded by the presence of numts. However, the total reads from each sample varied considerably, ranging from 16,156 to 2,042,234 mapped reads. Sample-to-sample variation in reads mapped can raise problems when designing experiments that require specific minimal read depths for each sample. We therefore sought to better equalize input DNA amounts for each sample in the next run. Toward this end, in our final run we more carefully normalized the input DNA amounts following adapter ligation (see Methods). This approach indeed resulted in better uniformity of total reads per sample (Table 2). Maximal mtDNA enrichment, in both the blood and the cell line, was again obtained from DC isolation followed by digest. We also included in NGS Run 3 cell-free DNA (cfDNA) from plasma. Although there is some degree of enrichment over baseline, the level does not approach that in the digested isolates.

**Table 2:** Summary of NGS Runs 2 and 3 for Cell lines and Blood Components.

Run	Sample	Total Reads	Reads Aligned (% of total)	Aligned to Mitochondrial Genome (% of aligned)	Fold Enrichment*
2	COLO 829 MB	1456822	1366821 (93.82%)	6168 (0.45%)	NA
2	COLO 829 DC	2042234	1880404 (92.08%)	105553 (5.61%)	NA
2	COLO 829 MB Digest	22922	19349 (84.41%)	1474 (7.61%) 0)	NA
2	COLO 829 DC digest	16156	13086 (81.00%)	8085 (**61.78%**)	**NA**
2	*ρ* ^0^ gDNA	854566	808326 (94.59%)	91 (0.01%)	0.02
2	1309 PBMCs gDNA	1998350	1877178 (93.94%)	10231 (0.55%)	1.00
2	1309 Buffy Coat MB	1391126	1260611 (90.62%)	16612 (1.32%)	2.42
2	1309 Buffy Coat DC	1681104	1550309 (92.22%)	60885 (3.93%)	7.21
2	1309 PBMCs Digest	260710	240976 (92.43%)	20009 (8.30%)	15.23
2	1309 Buffy Coat DC Digest	41528	38090 (91.72%)	9488 (**24.91%**)	**45.70**
3	COLO 829BL gDNA	441140	410072 (92.96%)	1208 (0.29%)	1.00
3	COLO 829BL MB	560102	512107 (91.43%)	2034 (0.40%)	1.35
3	COLO 829BL DC	1783560	1679059 (94.14%)	29357 (1.75%)	5.94
3	COLO 829BL gDNA Digest	2276262	2087881 (91.72%)	13817 (0.66%)	2.25
3	COLO 829BL MB Digest	1122872	920826 (82.01%)	123541 (13.42%)	45.54
3	COLO 829BL DC Digest	370496	309341 (83.49%)	107139 (**34.63%**)	**117.57**
3	*ρ* ^0^ gDNA	723274	628386 (86.88%)	72 (0.01%)	0.04
3	1312 PBMCs gDNA	1181402	1106903 (93.69%)	1698 (0.15%)	1.00
3	1312 Buffy Coat MB	664134	616025 (92.76%)	13786 (2.24%)	14.59
3	1312 Buffy Coat DC	566140	537704 (94.98%)	4667 (0.87%)	5.66
3	1312 PBMCs Digest	503418	472681 (93.89%)	18227 (3.86%)	25.14
3	1312 Buffy Coat DC Digest	431816	385146 (89.19%)	125817 (**32.67%**)	**212.95**
3	1312 cfDNA	636960	596453 (93.64%)	4365 (0.73%)	4.77

*Fold enrichment is computed over unenriched gDNA from the the same sample.

NA: gDNA from COLO829 not included on this run. Results from DC digest are shown in bold.

## Discussion

In this study, we have compared various approaches to preparing mtDNA from human blood and cell lines for NGS. Our conclusion here is that, among the various strategies examined, the optimal is differential centrifugation isolation followed by exonuclease digest. In our hands, this strategy yielded ~36% mtDNA reads in blood and ~37–62% in cell lines, which corresponds to hundreds-fold enrichment over baseline. This level of enrichment exceeds that which was previously reported in murine cell lines [14] (22.47% mtDNA reads) for MB isolation.

Deep sequencing of mtDNA is necessary to detect low-level heteroplasmic variants, and to accurately estimate relative abundances of different variant alleles in the cell. Accuracy has substantial clinical importance. For instance, the impact of a deleterious mtDNA mutation can be very closely tied to its allelic abundance in patient cells [15].

If sequencing is performed on total genomic human DNA from lymphocytes, only ~ 0.2% of the reads are expected to be mitochondrial. Therefore, if 100X average coverage of the mitochondrial genome is desired, it would be necessary to generate over 1.5 billion bases of sequence. Enriching the sample for mtDNA therefore allows far more samples to be multiplexed on the same sequencing run, resulting in substantial cost savings. Minimizing nDNA content also reduces the risk of numt-induced artifacts. Long-range PCR clearly provides the greatest level of enrichment, approaching 100% mtDNA reads. However, as we have shown, sequencing artifacts can arise even when using a high-fidelity polymerase. These artifacts can take the form of false variants, or shifts in relative allelic abundance at heteroplasmic sites. Furthermore, performing long-range PCR can be difficult in settings where DNA quantity is scant. This problem is particularly exacerbated when sequencing from blood, in which some fractions have extremely low DNA content.

There are a number of other potential avenues for mtDNA enrichment. As an alternative to amplification using long-range PCR, we have investigated the use of rolling circle isothermal amplification with random primers [16]. However, large amounts of primer-dimers were generated in the no-template control (water), indicating that many of the reads in the NGS run would be by-products of the amplification process. Another approach worth exploring would be a refinement of the isolation, where the outer membrane is stripped off prior to DNA extraction and sequencing. This approach is motivated by the observation that, despite mitochondrial isolation, there still is substantial nDNA contamination. It is therefore possible that DNA is adhering to the mitochondrial outer membrane. This phenomenon has been observed for RNA [17], and was ameliorated by removing the outer membrane to create mitoplasts. In any case, an ideal protocol would yield near-100% mtDNA reads without any amplification step. However, even at the enrichment levels attained in the current study, approximately 100 samples could be sequenced on a single MiSeq run at an average coverage of 1000X.

Although next-generation sequencing costs continue to decrease, studies involving large numbers of individuals still face limitations when using NGS technology. Mitochondrial DNA sequencing has the economic advantage of an extremely small genome, but *a priori* separation of the mitochondrial genome from the nuclear genome remains non-trivial. PCR can induce sequence artifacts and may not be possible if the sample has low DNA abundance.

In this study, we have compared various approaches to isolating mtDNA from blood and cell lines, and have concluded that the optimal approach entails differential centrifugation followed by exonuclease digest. This latter step is a novel application, as the digest is typically used to remove contaminating bacterial DNA from plasmid preparations. Our approach enriches mtDNA content hundreds-fold over baseline, requires only 1 ng of starting material, and does not involve PCR prior to library preparation. High throughput sequencing has much promise to facilitate a deeper understanding of human mitochondria-related diseases. It is therefore crucial that methods to ensure the most efficient and accurate assessment of mtDNA content continue to be developed.

## Supporting Information

S1 Table Alignment statistics with and without duplicate removal. (XLSX)

S2 Table Estimated Copy number of (*MT-ND1*)-*RPPH1* (2^-ΔCt^). (PDF)
