# Correction: PCR-Free Enrichment of Mitochondrial DNA from Human Blood and Cell Lines for High Quality Next-Generation DNA Sequencing

**DOI:** 10.1371/journal.pone.0156884

**Published:** 2016-05-31

**Authors:** Meetha P. Gould, Colleen M. Bosworth, Sarah McMahon, Sneha Grandhi, Brian T. Grimberg, Thomas LaFramboise

The fifth author’s surname is spelled incorrectly. “Grimerg” should instead be spelled “Grimberg.” The correct name is: Brian T. Grimberg. The correct citation is: Gould MP, Bosworth CM, McMahon S, Grandhi S, Grimberg BT, LaFramboise T (2015) PCR-Free Enrichment of Mitochondrial DNA from Human Blood and Cell Lines for High Quality Next-Generation DNA Sequencing. PLoS ONE 10(10): e0139253. doi:10.1371/journal.pone.0139253.
