# Supplementary material for: PCR-Free Enrichment of Mitochondrial DNA from Human Blood and Cell Lines for High Quality Next-Generation DNA Sequencing
Source: PLoS One. 2015 Oct 21;10(10):e0139253. doi: 10.1371/journal.pone.0139253 (PMC4619561; doi:10.1371/journal.pone.0139253)
Supplement: S2 Table — (PDF) [file pone.0139253.s002.pdf]

S2. Table Estimated mitochondrial copy number from human gDNA using qPCR.

	(*MT-ND1)-RPPH1* ($2^{-\Delta Ct}$)			
	**Pooled gDNA**		**1312 PBMCs gDNA**	
**Nuclear copy number expected from dilution***	**Mean**	**(s.d.)**	**Mean**	**(s.d.)**
**100**	352.67	(87.74)	301.97	(130.18)
**1000**	382.97	(66.59)	399.61	(76.07)
**10000**	343.81	(39.08)	498.44	(147.50)
**100000**	240.02	(110.07)	281.08	(48.14)
**Average**	329.87		370.28	

*** http://www3.appliedbiosystems.com/cms/groups/mcb_marketing/documents/generaldocuments/cms_042486.pdf**
